# Editorial: The safety assessment of novel foods and new nutrient sources

**DOI:** 10.3389/ftox.2026.1965747

**Published:** 2026-08-31

**Authors:** Luisa Pozzo, Maria Glymenaki

**Affiliations:** 1 Institute of Agricultural Biology and Biotechnology, National Research Council (CNR), Pisa, Italy; 2 European Food Safety Authority, Parma, Italy

**Keywords:** allergenicity, food safety assessment, new approch methods (NAMs), novel foods, toxicology

Scientific and technological advances in the food sector along with growing interest in sustainable dietary sources with high nutritional value are driving food innovation and the production of novel food products. Novel foods can derive from unconventional sources like microorganisms, algae, fungi, plants, or animals, be chemically synthesized, or be produced by innovative processes. The growing diversity and complexity of novel foods introduces new challenges for their safety assessment. A comprehensive analysis of their chemical composition, production process, intended uses and use levels, together with their toxicological evaluation is necessary to establish safety for human consumption. The nutritional content also needs to be considered as novel foods providing alternatives to traditional dietary sources of animal and plant origin must provide a similar or adequate balance of nutrients. Additionally, they may introduce new food allergens into the diet calling for assessment of their allergenic potential.

This Research Topic comprises seven contributions, including original research articles, and one review, highlighting recent advances in the toxicological and allergenicity safety assessment of novel foods and new nutrient sources.

An important theme that emerged from this Research Topic is the toxicological safety assessment of novel foods including genotoxicity and repeated dose toxicity. Schreppler et al., employed a standard battery of *in vitro* genotoxicity tests (bacterial reverse mutation assay, *in vitro* mouse lymphoma TK gene mutation assay, and *in vitro* micronucleus assay) to assess the genotoxic potential of short-, medium-, and long-chain triacylglycerides. This synthetic mixture of triacyglycerides showed no evidence of mutagenic, clastogenic, or aneugenic activity. Overall, the available data indicated that this novel food did not exhibit genotoxic potential under the tested conditions supporting its safety as a dietary fat ingredient. Mahadevan et al., demonstrated the safety of *Hericium erinaceus* and *Trametes versicolor* mushroom powders in acute and sub-chronic oral toxicity studies, as well as in *in vitro* and *in vivo* genotoxicity assays.

Long-term safety of the consumption of novel foods was further addressed by Punvittayagul et al., who evaluated formulated Thai rice instant granules containing turmeric extract and *Phyllanthus emblica* fruit pulp in a 6-month repeated-dose oral toxicity study and revealed no adverse effects. Given the presence of plant-based bioactive compounds, the expression profile of hepatic antioxidant genes was examined and found to be upregulated. Especially their work on molecular docking to identify binding affinity interactions between major bioactive compounds and key antioxidant enzymes provides an example of the application of mechanistic toxicology to gain insights into the antioxidant potential of these bioactive constituents present in the granules.

Mechanistic insights into the antioxidant potential of bioactive compounds in novel foods were further provided by Giambastiani et al., who investigated the effects of dietary supplementation with the microalga *Chlorella vulgaris* in an animal study. The findings showed elevated activity of hepatic xenobiotic-metabolizing cytochrome P450 (CYP) enzymes together with antioxidant and detoxification enzymes without signs of liver and kidney toxicity. The antioxidant potential of *C. vulga*ris was supported by compositional data indicating its richness in phenolic compounds and carotenoids. Additionally, their work illustrated anti-inflammatory and antioxidant effects *of C. vulga*ris in a murine model of chronic pulmonary inflammation. These findings further support the need for comprehensive toxicological evaluations on the long-term effects of novel foods containing bioactive compounds with pharmacological activities.

Another important theme emerging from this Research Topic concerns the complexity of the safety assessment in cases of products of cellular agriculture and the increasing importance of the application of New Approach Methodologies (NAMs). Felicianna et al., investigated the chemical characterization and toxicity of plant cell cultures from scurvy grass (*Cochlearia danica*) and rowan (*Sorbus aucuparia*) and demonstrated that they are nutritionally comparable to other berry cell lines without toxic effects. By applying proteomics analysis to identify potential allergens, the study showcases how NAMs are incorporated in the allergenicity safety assessment of novel foods. Their findings also emphasized the need for further work to evaluate the outcomes of proteomics, and the effects from phytohormone accumulation used in the growth media on the quality and safety of these plant cell culture foods.

The allergenicity assessment of novel foods was also addressed by Calcinai et al., who performed protein profiling of chia seeds (*Salvia hispanica*) followed by *in silico* homology analysis between identified chia peptides and sesame protein sequences. The potential cross-reactivity with characterised linear epitopes from sesame allergens was further corroborated *in vitro* by IgE-binding assays using sera from sesame-allergic individuals. This study highlights the challenges faced in the assessment of the allergenic potential of novel proteins including the lack of comprehensive protein sequence data and the insufficient characterization of potential allergenic epitopes.

Complementing these studies, Laganaro et al. reviewed the data requirements for allergenicity safety assessment of novel foods within the EU framework, identifying key uncertainties and research needs. The assessment should first consider the nature of the novel food, whether proteins are involved in its production, and whether its source is known to be allergenic. Literature findings, together with protein digestibility and stability data, also inform the evaluation of its allergenic potential. Where uncertainty remains, a tiered approach is applied to investigate potential cross-reactivity with known allergens, encompassing *in silico*, *in vitro* and, where necessary, *in vivo* approaches. The review underscores the value of integrating bioinformatic predictions with experimental validation to strengthen the evidence base for allergenicity assessment, while also highlighting the need for consensus on the interpretation of results, standardised and validated methods, and development of *de novo* sensitization assays.

Addressing limitations and data gaps in the safety assessment of novel foods is crucial for food innovation without compromising public health. The next-generation of safety assessment is expected to increasingly integrate NAMs and AI, while addressing the need for method standardisation and validation to support fit-for-purpose regulatory decision-making. Overall, the contributions assembled in this Research Topic reflect the breadth of approaches for toxicological and allergenicity assessment and illustrate the ongoing evolution towards more mechanistic, predictive and biologically relevant methodologies.

## Author disclaimer

MG is employed with the European Food Safety Authority (EFSA) in the Nutrition and Food Innovation Unit that provides scientific and administrative support to the Panel on Nutrition, Novel Foods, and Food Allergens, in the area of Novel Foods. However, the present work is published under the sole responsibility of the author and may not be considered as an EFSA scientific output. The positions and opinions presented in this article are those of the author alone and do not necessarily represent the views or scientific work of EFSA.

